# The development of the fear of earthquake scale: validity and reliability study in Türkiye after the 2023 earthquake

**DOI:** 10.1186/s40359-023-01477-9

**Published:** 2023-12-07

**Authors:** Tuğba Sarı, Arzu Taşdelen-Karçkay, Şule Tarcan

**Affiliations:** Department of Guidance and Psychological Counselling, Akdeniz University, Antalya, Turkey

**Keywords:** Fear of Earthquake Scale, Psychometric properties, Resilience, Earthquake-related fears

## Abstract

**Background:**

In 2023, Türkiye experienced a significant earthquake disaster that profoundly impacted 11 provinces. The enduring consequences of these earthquakes on daily life triggered widespread fears and anxieties in society, leading to scholarly investigations in this field.

**Objective:**

The primary objective of this study was to create and evaluate the psychometric properties of the Fear of Earthquake Scale (FES), a modified adaptation of the Fear of COVID-19 Scale (FCV-19 S), tailored to measure earthquake-related experiences in Türkiye.

**Methods:**

A total of 315 Turkish adult participants (106 men, 209 women), with a mean age of 37.71 years, completed the FES, along with the Brief Psychological Resilience Scale (BPRS). Psychometric analyses included confirmatory factor analysis as well as the evaluation of alternative factor structures, internal consistency, convergent validity, and criterion validity with respect to resilience.

**Results:**

The findings indicate that the Turkish version of the Fear of Earthquake Scale has strong psychometric properties in terms of validity and reliability. After assessing various factor structures, it was observed that the two-factor model which represents the emotional and somatic response to fear, exhibited the best-fit values The Cronbach’s alpha coefficients were calculated as 0.89 for the overall FES, 0.84 for the emotional subscale and 0.86 for the somatic subscale, indicating high internal consistency. Additionally, the negative correlation between resilience and the FES supports the criterion validity of the scale, and multi-group confirmatory factor analyses proved that measurement invariance held across genders and whether they experienced an earthquake or not for all groups. Furthermore, the results of the study revealed that women and individuals with prior earthquake experience reported higher levels of fear of earthquakes.

**Conclusions:**

The FES emerged as a reliable and valid tool for assessing earthquake-related fears among the Turkish population.

## Introduction

Earthquakes, characterized by their suddenness and lack of forewarning, present an immediate and significant danger to individual’s lives and well-being, with the extent of their impact determined by their intensity [1]. Numerous large-scale earthquakes have occurred in different regions of Türkiye throughout history. The most recent major earthquake occurred on February 6, 2023, affecting 11 cities in the eastern and southeastern regions of Anatolia. This severe earthquake resulted in the collapse of thousands of buildings and the loss of tens of thousands of lives [2]. Many people have lost loved ones, and many more have been left homeless. Even more than six months after the event, many earthquake victims are still dealing with severe psychological issues. The fear caused by disasters weakens one’s sense of control and damages one’s confidence in themselves and their environment [3–5]. Even if individuals are not directly involved, earthquakes have a substantial detrimental impact on people’s lives and psychological health [6].

The traumatic impacts of such occurrences may spread outside the disaster zone, and symptoms may linger for a long time after the incident [7]. For instance, the 1988 Armenia earthquake had such a significant effect that even skilled international rescue workers still had trouble sleeping nine months after they returned home [8]. Additionally, it has been observed that American teenagers of Armenian descent, who are geographically distant from the disaster area, can exhibit posttraumatic symptoms such as guilt, psychological numbness, and anxiety when they witness news coverage of the event on television [9]. These findings highlight the far-reaching psychological effects of earthquakes and demonstrate that individuals can be affected even from a distance due to their emotional connections or exposure to media coverage of the disaster.

### Fear of earthquakes and its impact on mental health

Numerous studies [10–12] investigating the psychological aftermath of earthquakes have consistently highlighted fear as the predominant emotional reaction. Research findings indicate that among those who have survived earthquakes, individuals experiencing elevated levels of fear are more prone to displaying symptoms associated with posttraumatic stress disorder [13]. Additionally, a study conducted by Şalcıoğlu et al. [14] among earthquake survivors in Türkiye revealed that the fear of future earthquakes and a decrease in perceived life control were strong predictors of PTSD. The fear of earthquakes has various psychological effects that contribute to the psychological distress experienced by survivors in the aftermath of an earthquake. These findings highlight the significance of the perceived fear of earthquakes in comprehending the psychological consequences of earthquake experiences [15].

Fear is an emotional response that emerges in the face of immediate and tangible danger [16]. It serves as an innate protective mechanism, alerting individuals to potential threats to their lives and well-being, thereby hindering the fulfillment of their needs. Fear can be triggered by various concrete dangers, including earthquakes, floods, droughts, the threat of war, the loss of loved ones, unemployment, assaults, or robberies. However, if individuals continue to experience persistent unease, worry, and restlessness even after the removal of these concrete dangers, it may indicate the development of anxiety disorders [17].

Extensive research indicates that individuals who harbor a high fear of earthquakes tend to experience adverse psychological conditions, including anxiety [18–20], depression, and restlessness [21, 22]. Moreover, they often exhibit a slower recovery following an earthquake [23]. Alongside psychological distress, individuals may also experience physiological reactions such as sleep disturbances, increased heart rate, and respiratory problems [19]. A study conducted after a disaster demonstrated that one year later, young survivors still experienced significant levels of fear related to vibrations (89.9%], new earthquakes (81.1%), loud noises (58.7%), and buildings (49.5%) and exhibited avoidance behaviors, such as avoiding going to school (26.5%) [9].

### Purpose of the current study

Conducting research on earthquake fear in diverse populations holds significant importance. Considering the unavailability of measurement tools specifically designed for assessing earthquake fear in Türkiye, the present study sought to develop the Fear of Earthquake Scale (FES). In pursuit of this goal, the study draws from the Fear of COVID-19 Scale (FCV-19 S), which was adapted and employed to measure earthquake fear in a Croatian sample [15]. The FCV-19 S, designed to examine fear responses related to the coronavirus, encompasses both somatic and emotional fear reactions [31]. This makes it an appropriate tool for evaluating earthquake-related fear, considering that individuals affected by earthquakes have reported both types of reactions [19]. Notably, studies have produced varying outcomes concerning the underlying factor structure of the FCV-19 S questionnaire. While certain research supports a one-factor structure [24–27], others unveil correlations of error variances among specific items [28–32]. As an alternative, a two-factor structure is proposed, with one factor capturing emotional aspects and the other somatic features of fear associated with COVID-19 [33–38]. These contrasting findings underscore the intricate and diverse nature of individuals’ responses to the fear of COVID-19.

Given the diverse findings in studies exploring factor structure-related scales such as the FCV-19 S, it becomes evident that understanding the complex nature of fear is crucial. With this perspective in mind, our study pursued two primary objectives. First, we aimed to construct and assess the psychometric properties of the Fear of Earthquake Scale (FES), which is a modified adaptation of the FCV-19 S [39], specifically designed to measure earthquake-related experiences in Türkiye. Second, we conducted a comprehensive comparative analysis, treating the single-factor and two-factor models of the FES as competing frameworks for understanding earthquake fear. Additionally, the investigation seeks to explore potential gender-based and prior earthquake experience-related variations in earthquake fear.

## Method

### Participants

The participants of the study consist of 315 adults aged 18 and above living in Türkiye who have experienced earthquake events and those who have not. The enrollment of these participants was accomplished through the utilization of the convenience sampling method. Among the participant group, 209 (66%) were female and 106 (34%) were male. There were 182 (58%) participants who had experienced earthquake events and 133 (42%) participants who had not. Furthermore, among the participants, 81 individuals (26%) fell within the age range of 18–29, 97 individuals (31%) fell within the age range of 30–39, 86 individuals (27%) fell within the age range of 40–49, 41 individuals (13%) fell within the age range of 50–59, and 10 individuals (3%) were 60 years old and above. The distribution of participants based on their perceived economic status as low-income, middle-income, and high-income was as follows: 32 individuals (10%) reported low-income, 260 individuals (83%) reported middle-income, and 23 individuals (7%) reported high-income (see Table 1).

**Table 1:** Demographic Characteristics of the Participants Note: Percentages may not total 100% due to the rounding

	N	Percentage (%)		
Gender				
Woman Man	209		66.3	
	106		33.7	
Age				
18-29	81		25.7	
30-39	97		30.8	
40-49	86		27.3	
50-59	41		13	
60 and above	10		3.2	
Educational Level				
Primary School	11		3.5	
Middle School	12		3.8	
High School	61		19.4	
Undergraduate	191		60.6	
Graduate	40		12.7	
Economic Status				
Low	32		10.2	
Middle	260		82.5	
High	23		7.3	
Experience an Earthquake				
Yes	182		57.8	
No	133		42.2	

### Instruments

#### Demographic information form

In line with the research objective, the researchers created a personal information form to collect demographic data. The form included five items related to gender, age group, educational background, perception of economic status, and prior experience with earthquakes.

#### Fear of Earthquake Scale (FES)

The FES was designed to assess the level of fear associated with earthquakes. We adapted the original items from the FCV-19 S [39] questionnaire by replacing the context of fear of COVID-19 with fear of earthquakes. The scale was modified to include expressions of fear related to earthquakes instead of COVID-19. It consisted of a total of 7 items and used a 5-point Likert scale (1 “strongly disagree” to 5 “strongly agree”). Higher scores on the scale indicated a higher level of fear of earthquakes. Sample items from the scale included statements such as “I am most afraid of earthquakes” and “My heart races or palpitates when I think about earthquakes.“ In the Croatian study conducted by Prizmic-Larsen et al. [15], an EFA was conducted to assess the validity of the FES. The results showed a unidimensional structure, with one dominant factor accounting for 58% of the variance in the FES scores, as indicated by an eigenvalue of 4.08. Information on the reliability and validity of the scale is presented in the results section of the current study.

#### Brief psychological resilience scale

The Brief Resilience Scale (BPRS), originally developed by Smith et al. [40] and later adapted by Doğan [41], is a Likert-type measurement tool comprising six items, each rated on a five-point scale. In the adaptation study, an exploratory factor analysis (EFA) was conducted, revealing a unidimensional structure that accounted for 54% of the total variance. The factor loadings ranged from 0.63 to 0.79, indicating strong associations between the items and the underlying factor. Additionally, a confirmatory factor analysis (CFA) was performed in this study to assess the goodness of fit for the one-dimensional structure. The results indicated favorable fit indices, including χ2 = 20.315, df = 6 (χ2/df = 3.38), the adjusted goodness of fit index (AGFI) = 0.97, the goodness of fit index (GFI) = 0.99, the comparative fit index (CFI) = 0.99, the root mean square error of approximation (RMSEA) = 0.058, and the root mean residual (RMR) = 0.015, suggesting that the model adequately represents the data. The item-total correlation coefficients ranged from 0.61 to 0.70, indicating moderate to strong relationships between individual items and the overall scale. Furthermore, the internal consistency of the scale was satisfactory, with a Cronbach’s alpha coefficient of 0.86, indicating high reliability. The Cronbach’s alpha value of the BPRS in the current study was 0.74.

### Procedure

To initiate the study on the scale by modifying the items of the Fear of COVID-19 Scale, we first contacted the authors of the FCV-19 S via email. In our communication, we requested permission to modify the items of the Fear of COVID-19 Scale to measure the fear of earthquakes. We obtained permission to adapt the scale by replacing the expressions associated with COVID-19 fear in the FCV-19 S with earthquake-related fear expressions. After replacing the COVID-19 fear expressions in each item of the FCV-19 S with earthquake-related fear expressions, the translation process from English to Turkish was initiated. The initial translation was conducted by the first author, who is proficient in both Turkish and English. Expert opinions were then obtained from three academics specializing in this field. Based on the feedback received, revisions were made to the scale items, and the final version was determined. The forward translations were compared by the initial and the second authors, leading to a reconciliation. The scale was then translated back into English by the first author. In the final phase, the three researchers reviewed each item in both Turkish and English. The authors’ university then authorized the submission of an application to the ethics committee. Before participating, all individuals were informed about the study’s objectives, and each participant provided informed consent.

Data collection for the study took place after a significant seismic event on February 6, 2023, which resulted in a 7.8 magnitude (Mw) earthquake. The data were collected from April to June. The Google Form was organized in the following order: Demographic Information Form, Brief Psychological Resilience Scale, and Fear of Earthquake Scale (FES). The researchers utilized social media platforms to share the online forms they had prepared with their social circles, as well as with undergraduate and graduate students. The snowball sampling technique was used to ensure a diverse participant tool, including individuals from various cities. The application link was specifically shared with volunteer participant groups in different cities, including those residing in earthquake-affected areas.

### Data analyses

All analyses, except for CFA were conducted using IBM SPSS Statistics 25. For CFA, IBM SPSS Amos 23 was employed. Before conducting validity and reliability tests, the normality assumptions of the scale items were checked and met. A CFA was performed to test the construct validity of the five-item single-factor model of the scale. Additionally, the criterion-related validity of the scale was examined by assessing its relationship with the Brief Psychological Resilience Scale.

CFA was conducted on different models for the FES in our study. These models include the one-factor structure model (M1), an alternative one-factor model with three correlated pairs of residuals (M2), a two-factor model with emotional response and somatic response factors (M3), and an alternative two-factor model with one correlated pair of residuals (M4). The chi-square (χ2) value divided by degrees of freedom (df) was calculated to evaluate the fit of the one-factor structure model [33]. A chi-square value divided by degrees of freedom less than 2 indicates excellent fit [42], while a range of 2–5 represents acceptable fit [43, 44]. The following fit indices were used to assess the fit of the models: the GFI, the CFI, the incremental fit index (IFI), and the RMSEA. The GFI, CFI, and IFI values between 0.90 and 0.95 and RMSEA values close to 0.06 indicate good model fit [45, 46]. The expected cross-validation (ECVI) and the Bayesian information criterion (BIC) values were used to compare single-factor and two-factor models. Smaller ECVI and BIC values indicate better model fit [47, 48]. Convergent validity was examined by calculating the average variance explained (AVE), with a criterion of AVE greater than 0.50. Cronbach’s alpha and corrected item correlation values were calculated to determine the reliability of the proposed model. Acceptable alpha values range from 0.70 to 0.95 [49, 50]. In general, for a good scale, corrected item-total correlations should fall within the range of 0.30 to 0.70 [51].

## Results

### Assumption testing

In this study, descriptive statistics were generated to identify the number of missing values for each variable in the dataset. No missing data were found. Z-scores were calculated, and a comprehensive analysis of histogram and box-plot visuals was conducted to determine both univariate and multivariate outliers, as well as to assess the normality of distributions. After this examination, three participants were identified as outliers and excluded from the study. The univariate skewness and kurtosis values suggest that the responses were relatively normally distributed [52]. The descriptive statistics for the items of the FES are presented in Table 2.

### Construct validity

Table 3 presents the fit indices for various models applied to the FES. The one-factor model (M1) CFA did not show an acceptable model fit. However, further analysis of the modification indices identified significant error correlations between specific items (items 1 and 2, 4 and 5, 6 and 7). These correlations suggested interdependence among the error terms of these items, contributing to the elevated χ2/df value. The results showed that this modified model can be considered acceptable, as indicated by the χ2/df value (4.527) which is below the recommended threshold of 5.

The two-factor model (M3) CFA demonstrated an acceptable model fit. Upon analyzing the modification indices, substantial error correlations were identified between item 1 and item 2. After analyzing the data with the modification, notable improvements were observed in the goodness-of-fit indicators, as indicated by the χ2/df value of 2.173 for the alternative two-factor model with one correlated pair of residuals (M4). The fit indices of the alternative two-factor model with one correlated pair of residuals (M4) support model fit.

**Fig. 1:**
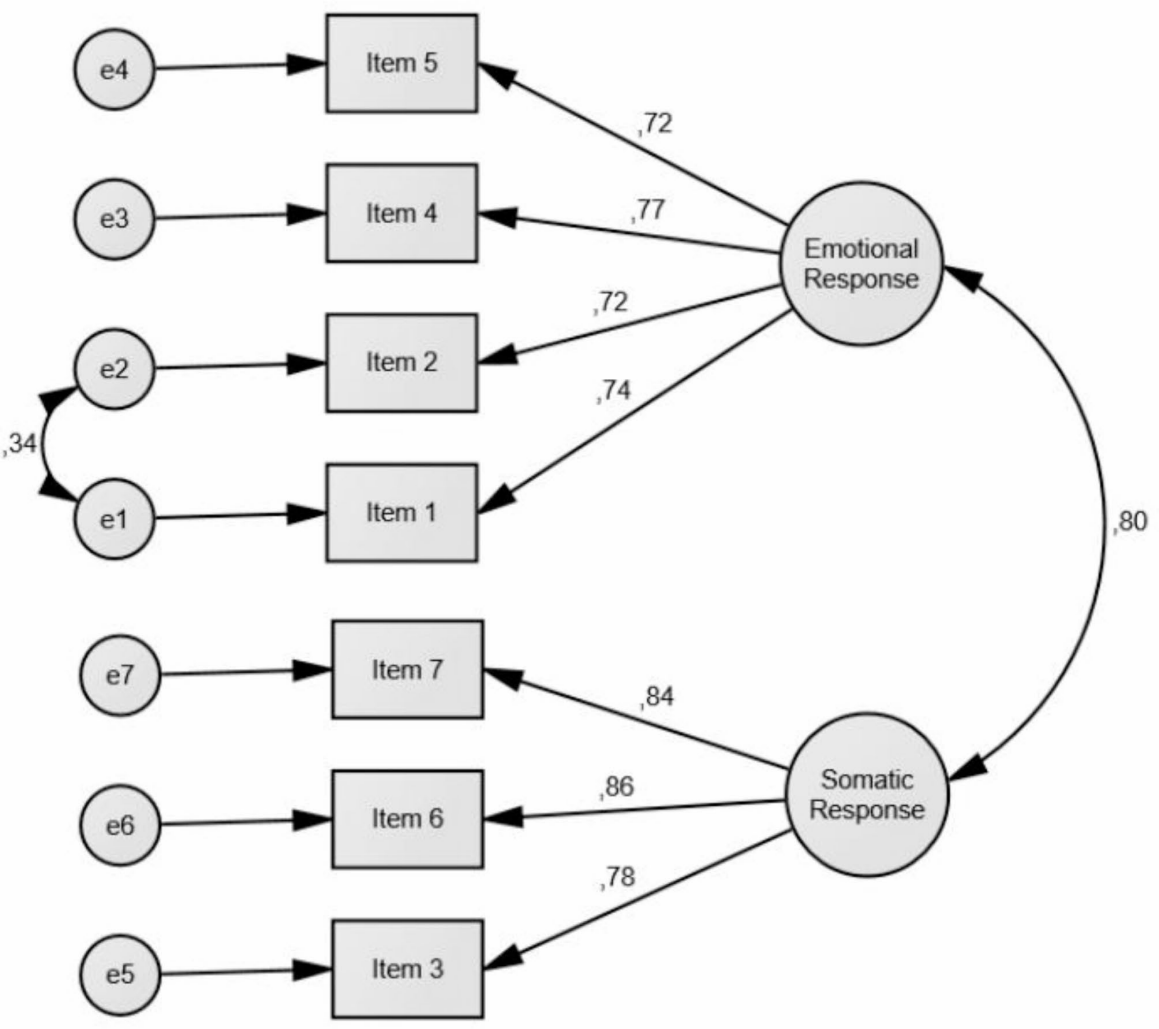
Confirmatory Factor Analysis Results for the Two-Factor Model of the Fear of Earthquake Scale (N = 315)

All measurement models were compared using ECVI and BIC values. Among them, the alternative two-factor model with one correlated pair of residuals (M4) demonstrated the lowest ECVI and BIC values (as presented in Table 3). As a result, the alternative two-factor model with one correlated pair of residuals (M4), encompassing both emotional (items 1, 2, 4, 5) and somatic (items 3, 6, 7) aspects of fear of earthquakes, was identified as the most suitable fit for the data (see Table 3; Fig. 1). Furthermore, the AVE value of the overall scale was determined to be 0.60, while it was found to be 0.54 for the emotional response subscale and 0.69 for the somatic response subscale. These findings further confirm the appropriateness of the proposed model fit in the context of the validity study.

**Table 2:** Confirmatory Factor Analysis of the FES for all measurement models

	χ2	df	χ2/sd	GFI	CFI	IFI	RMSEA	ECVI	BIC
M1: One − factor model	152.995	14	10.928	0.862	0.880	0.880	0.178	0.576	233.531
M2: Alternative one-factor model with 3 correlated pairs of residuals	49.795	11	4.527	0.955	0.966	0.929	0.106	0.267	147.588
M3: Two-factor model	46.051	13	3.542	0.960	0.971	0.972	0.090	0.242	132.339
M4: Alternative two-factor model with 1 correlated pair of residuals	26.073	12	2.173	0.978	0.988	0.988	0.061	**0.185**	**118.114**

### Reliability analyses

To evaluate the measuring power of each item in the FES and assess item reliability, item-total statistics were computed. The results of the corrected item correlations are presented in Table 2, with values ranging from 0.63 to 0.77. The overall Cronbach’s alpha coefficient for the FES scale was determined to be 0.89, indicating a high level of internal consistency. Furthermore, the emotional response factor exhibited a Cronbach’s alpha coefficient of 0.84, while the somatic response factor demonstrated a Cronbach’s alpha coefficient of 0.86. Additionally, composite reliability (CR), which measures internal consistency, was assessed and yielded values of 0.91 for the total scale, 0.83 for the emotional response subscale, and 0.87 for the somatic response subscale. These findings provide further evidence supporting the reliability of the FES subscales.

**Table 3:** Descriptive Statistics of the FES Items, Factor Loads, and Adjusted Item Total Correlation Scores for an alternative two-factor model with 1 correlated pair of residuals (M4)

Items	M	SD	Corrected Item Correlation	Skewness	Kurtosis	Factor Loads^a^
1. I am most afraid of earthquakes.	3.63	0.069	0.712	−0.516	−0.663	0.744
2. It makes me uncomfortable to think about earthquakes.	3.72	0.074	0.691	−0.709	−0.659	0.721
3. My hands become clammy when I think about earthquakes.	2.19	0.074	0.697	0.856	−0.386	0.776
4. I am afraid of losing my life because of an earthquake.	3.16	0.081	0.667	−0.102	−1.292	0.770
5. When watching news and stories about earthquakes on social media, I become nervous or anxious.	3.66	0.072	0.631	−0.608	−0.726	0.722
6. I cannot sleep because I’m worrying about getting an earthquake.	2.14	0.071	0.770	0.834	−0.353	0.857
7. My heart races or palpitates when I think about getting an earthquake.	2.41	0.077	0.752	0.593	−0.809	0.841

### Criterion validity

We conducted a bivariate correlational analysis to examine the relationship between the overall score of the FES and its two subscales (emotional responses and somatic responses) as well as the overall score of the Brief Resilience Scale (BRS) to assess criterion validity. The correlation analysis revealed a statistically significant negative association between the FES and BRS total scores, with a correlation coefficient of − 0.383 (*p* < .001). The emotional response and somatic response subscales also showed negative correlations with the BRS. The correlation for the somatic response subscale was − 0.324 (*p* < .001), while it was − 0.373 (*p* < .001) for the emotional response subscale.

### Configural invariance across groups

In our study, we investigated whether the two-factor structure of the FES could adequately describe the fear of earthquakes across different groups using the configural invariance model (M4). For gender groups, the results showed that χ2/df = 1.42, AGFI = 0.93, GFI = 0.97, CFI = 0.99, RMSEA = 0.036, and RMR = 0.026. For earthquake experience, the results showed that χ2/df = 2.37, AGFI = 0.90, GFI = 0.97, CFI = 0.97, RMSEA = 0.064, and RMR = 0.37. Additionally, all factor loadings reached a significant level (*p* < .05) for all groups. In summary, these findings suggest that the two-factor structure of fear of earthquakes adequately explains the FES in both gender and earthquake experience groups.

### Group differences: gender and earthquake experience

We also examined potential gender and earthquake experience differences in our study. The results revealed significant differences in both gender and earthquake experience. Specifically, females reported a significantly higher overall FES score (M_female_ = 21.608; SD_female_ = 6.808) compared to males (M_male_ = 19.519; SD_male_ = 7.666; *t* (2.465); *p* = .014; *d* = − 0.294). Furthermore, females exhibited a higher usage of emotional responses to fear (M_female_ = 14.665; SD_female_ = 4.045) than males (M_male_ = 13.179; SD_male_ = 4.712; *t* (2.91); *p* = .006; *d* = − 0.347).

In terms of earthquake experience, we found statistically significant differences in the somatic response subscale of the FES. Participants who had experienced earthquakes (M_had_experienced_ = 7.121; SD_had_experienced_ = 3.644) scored higher on the somatic response subscale compared to those who had not experienced earthquakes (M_did_not_have_experienced_ = 6.218; SD_did_not_have_experienced_ = 3.208; *t* (2.328), *p* = .021; *d* = − 0.260). These findings indicate that individuals who have experienced earthquakes report a significantly higher frequency of somatic responses to fear compared to those who have not experienced earthquakes.

## Discussion

Türkiye is a region that experiences frequent earthquakes, posing a continuous threat to its residents. The recent earthquake in February 2023 had a profound impact on densely populated residential areas in southern Türkiye and on numerous individuals directly or indirectly affected by this traumatic life event. The objective of this study was to develop a valid scale specifically tailored for the Turkish population to measure earthquake fear. Drawing inspiration from the Fear of COVID-19 Scale (FCV-19 S), we designed the Fear of Earthquake Scale (FES) which incorporates relevant items capturing various aspects of fear responses related to earthquakes.

In this study, we investigated the measurement models of the FES and explored its psychometric properties. Four different models were evaluated using a competing model approach. These models included the one-factor structure model proposed by Ahorsu et al. [39], an alternative one-factor model with three correlated pairs of residuals, a two-factor model with emotional response and somatic response factors, and an alternative two-factor model with one correlated pair of residuals. Our findings show that the most suitable model is a different two-factor model with only a correlated set of residuals. This model represents the FES accurately and robustly, despite the widely accepted one-factor model with one correlation. Our findings affirm the literature’s two-factor structure model of the FCV-19 S [33–38].

Prizmić-Larsen et al. [15] used the FCV-19 S as a basis to develop the Fear of Earthquake Scale in Croatia. They modified items and conducted an EFA for validation. The EFA showed a one-factor model with six correlated residual pairs. The FES development, stemming from the FCV-19 S modification, yielded diverse outcomes internationally, echoing the FCV-19 S results. Such findings contribute to the growing body of knowledge regarding fear and its universality across different crisis events. Further FES research on different populations and disasters can enhance cross-cultural insights.

The item-total statistical analysis for the FES revealed strong corrected item-total correlations for all items, ranging from 0.63 to 0.77. These values indicate a solid relationship between each item and the total scale score, confirming the measuring power of the items in capturing earthquake fear responses effectively. Furthermore, the internal consistency of the FES was assessed using Cronbach’s alpha coefficient, which demonstrated high reliability for the overall scale (α = 0.89). This result closely resembles the findings reported by Prizmić-Larsen et al. [15] in Croatia (α = 0.90).

Criterion validity was evaluated by examining the associations between the FES total scores and its subscales (emotional and somatic) and the Brief Resilience Scale (BRS) total score. The results indicated a significant negative link between the FES and BRS scores, suggesting that higher FES scores were associated with lower resilience. This supports the FES’s criterion validity, capturing fear responses and resilience levels. Similar findings were found in the Croatian study Prizmić-Larsen et al. [15]. Although limited prior research has explored the associations of the new FES scale, previous studies on the fear of earthquakes consistently showed associations in the same direction with various psychological outcomes [1, 53].

In this study, we also investigated the differences in earthquake fears between individuals based on two variables: gender and earthquake experience. The results highlighted significant variations in both gender and earthquake experience groups, offering insights into their influence on coping strategies. Regarding gender disparities, our findings indicated that females reported more earthquake fear and higher emotional fear response levels than males. Gün Çinği and Yazgan [54], Karanci et al. [55], Prizmić-Larsen et al. [15], and Sumer et al. [7] all found consistent evidence supporting the notion that women experience higher distress levels following an earthquake event. Brıni et al. [56] discovered that over February 6, 2023, the Türkiye Earthquake, women displayed higher levels of death grief, self-criticism, and inadequate mental well-being than men. These results indicate that gender matters in post-earthquake psychological reactions, with women experiencing more distressing emotional effects. These results show the importance of taking gender-related issues into account while managing crises and the aftermath of earthquakes, as Krishnaraj [57] demonstrated following the Latur earthquake in India. These gender-based differences are important for mental health interventions and therapy [58].

The study’s findings also demonstrated that individuals who had experienced an earthquake responded to fear considerably more somatically than those who had not. This emphasizes the significance of physical responses to fear associated with earthquakes. Increased heart rate, sweating, shaking, and a sense of restlessness or unease are physical signs of dread and anxiety brought on by earthquakes [19, 59, 60]. The study’s findings are in line with earlier studies that have shown how traumatic experiences such as earthquakes can have an ongoing impact on people’s fear reactions.

### Limitations

Although the Turkish version of the FES has shown promise in terms of validity and reliability, further evaluation and development in different populations and circumstances are necessary to improve its generalizability. Future research could explore how the FES relates to real earthquake experiences and evaluate its sensitivity to changes in anxiety levels over time. The lack of test-retest repeatability is a limitation of this study, which could be addressed in future studies to determine the consistency and reliability of the FES.

### Conclusions and implications

In conclusion, despite certain limitations, our study successfully created the Fear of Earthquake Scale, a useful instrument for evaluating earthquake-related fear in seismically active regions like Türkiye. The validity and reliability of the FES are supported by our findings, making it an effective tool for comprehensively assessing earthquake fear. The integrated and reliable two-factor structure of the FES provides a comprehensive understanding of how people respond to earthquake fear. Researchers can utilize the FES to gain insights into both the physiological and emotional reactions associated with earthquake fear, aiding in the selection of appropriate intervention strategies for those affected by earthquakes. The FES is a valuable resource for researchers and professionals in mental health care, disaster preparedness, and community resilience development in Türkiye. Its applicability extends to studies conducted across different cultures, providing a broader perspective on coping with earthquake fear.

## Data Availability

The data that supports the findings of the current study are available from the corresponding author upon reasonable request.
